# RANKL synthesized by articular chondrocytes contributes to juxta-articular bone loss in chronic arthritis

**DOI:** 10.1186/ar3884

**Published:** 2012-06-18

**Authors:** Maria J Martínez-Calatrava, Ivan Prieto-Potín, Jorge A Roman-Blas, Lidia Tardio, Raquel Largo, Gabriel Herrero-Beaumont

**Affiliations:** 1 Bone and Joint Research Unit, Service of Rheumatology, IIS Fundación Jiménez D237;az, Universidad Autónoma, Av. Reyes Católicos 2, 28040, Madrid, Spain

## Abstract

**Introduction:**

The receptor activator nuclear factor-kappaB ligand (RANKL) diffuses from articular cartilage to subchondral bone. However, the role of chondrocyte-synthesized RANKL in rheumatoid arthritis-associated juxta-articular bone loss has not yet been explored. This study aimed to determine whether RANKL produced by chondrocytes induces osteoclastogenesis and juxta-articular bone loss associated with chronic arthritis.

**Methods:**

Chronic antigen-induced arthritis (AIA) was induced in New Zealand (NZ) rabbits. Osteoarthritis (OA) and control groups were simultaneously studied. Dual X-ray absorptiometry of subchondral knee bone was performed before sacrifice. Histological analysis and protein expression of RANKL and osteoprotegerin (OPG) were evaluated in joint tissues. Co-cultures of human OA articular chondrocytes with peripheral blood mononuclear cells (PBMCs) from healthy donors were stimulated with macrophage-colony stimulating factor (M-CSF) and prostaglandin E_2 _(PGE_2_), then further stained with tartrate-resistant acid phosphatase.

**Results:**

Subchondral bone loss was confirmed in AIA rabbits when compared with controls. The expression of RANKL, OPG and RANKL/OPG ratio in cartilage were increased in AIA compared to control animals, although this pattern was not seen in synovium. Furthermore, RANKL expression and RANKL/OPG ratio were inversely related to subchondral bone mineral density. RANKL expression was observed throughout all cartilage zones of rabbits and was specially increased in the calcified cartilage of AIA animals. Co-cultures demonstrated that PGE_2_-stimulated human chondrocytes, which produce RANKL, also induce osteoclasts differentiation from PBMCs.

**Conclusions:**

Chondrocyte-synthesized RANKL may contribute to the development of juxta-articular osteoporosis associated with chronic arthritis, by enhancing osteoclastogenesis. These results point out a new mechanism of bone loss in patients with rheumatoid arthritis.

## Introduction

Rheumatoid arthritis (RA) is a chronic disease characterized by both synovial and systemic inflammation, with primary joint involvement. The intense inflammatory process seen in the disease is the most important risk factor for progressive destruction of extracellular matrices of articular cartilage and bone in joints affected by RA [1-3]. Three principal forms of bone loss have been described in patients with RA: focal bone erosions, juxta-articular bone loss, and systemic bone loss [4]. Of these, juxta-articular bone loss represents a common and early feature of RA that affects the trabecular bone adjacent to the inflamed joint [5]. In spite of this, the pathogenesis of juxta-articular bone loss in RA has not yet been fully elucidated, mainly because of the difficulties in obtaining appropriate human samples for study. Nevertheless, the loss of periarticular bone in RA has been associated with dysregulation of bone remodeling, which is redirected towards the predominance of resorption activity over formation [6].

Different hormones, cytokines and chemokines produced by the inflamed synovial membrane have been reported to be involved in juxta-articular osteoporosis in RA [4]. Also, the pannus directly infiltrates osseous tissue contributing to periarticular bone loss [7]. Certainly, juxta-articular bone loss is related to the intensity of inflammatory response in the affected joint [2-7]. This fact is observed not only in RA, but also in other arthritides associated with a high degree of inflammation [8,9]. Macrophages differentiate into bone-resorbing osteoclasts in zones of contact between the inflamed synovium and subchondral bone in RA in the presence of a crucial factor, the receptor activator of nuclear factor-κB ligand (RANKL) [10,11].

RANKL is markedly involved in osteoclastogenesis, osteoclast migration, adherence to bone, and apoptosis regulation due to its binding to the receptor activator of nuclear factor-κB (RANK) [12], which is expressed on osteoclast precursors and mature osteoclasts. A study by Pettit, *et al *showed that tumor necrosis factor-related activation-induced cytokine (TRANCE) knockout mice were deficient in osteoclasts and protected from bone loss in a serum transfer model of arthritis, demonstrating *in vivo *the importance of RANKL and osteoclastogenesis in bone loss associated with RA [13]. The pro-osteoclastogenic actions of RANKL are physiologically regulated by osteoprotegerin (OPG), a soluble non-signalling receptor for RANKL [14]. Indeed, OPG competitively inhibits RANKL binding to its receptor RANK. In healthy joints, RANKL expression has been described in bone lining cells of osteoblast lineage, synovial T cells, and chondrocytes [9,15-19], whereas in inflamed arthritic joints, it is detected in synovial fibroblasts, T and B cells, osteoclasts, and chondrocytes [6,20-23].

Proinflammatory cytokines such as tumor necrosis alpha (TNF-α), interleukin (IL)-1β, IL-6, IL-7, and IL-17, as well as macrophage-colony stimulating factor (M-CSF), parathyroid hormone (PTH), 1,25-dihydroxycholecalciferol [1,25(OH)_2_D_3_], and prostaglandin E_2 _(PGE_2_) increase RANKL synthesis [3,4,24,25]. More recently, transcriptional repressors that suppress RANKL-induced gene expression and osteoclast differentiation have been described, including IL-4/IL-13 and granulocyte-macrophage colony stimulating factor (GM-CSF), IL-10, IL-27, interferon-γ, TNF apoptosis related inducing ligand (TRAIL), IL-12, IL-18, IL-6, and toll-like receptors [26]. Thus, the extent of bone destruction in inflammatory arthritis is determined by the balance between osteoclastogenic and anti-osteoclastogenic factors, with the relevant biological mediation of RANKL.

In addition to membrane-bound RANKL in osteoblasts [27], RANKL secreted by synovial cells actively promotes bone destruction in chronic inflammatory arthritis [21,23]. Hence, high local RANKL concentrations may lead to increased osteoclastogenesis at the bone-pannus interface. However, scarce attention has been given to the potential role of the RANKL expressed by chondrocytes in the pathogenesis of RA-related juxta-articular bone loss, although it has been reported to diffuse from the cartilage to subchondral bone in human osteoarthritis (OA) [28]. Accordingly, we hypothesized that RANKL produced in articular cartilage might contribute to juxta-articular bone loss in chronic arthritis.

Animal models of RA offer a valuable opportunity to enhance our understanding of the pathogenic mechanisms underlying juxta-articular bone loss in the disease. To optimize this potential, we characterized an experimental model of chronic arthritis that represents a more intense and destructive version of the well-established antigen-induced arthritis (AIA) [29-31]. This inflammatory arthritis is accompanied by severe juxta-articular bone loss, as estimated by x-ray and bone mineral density (BMD). Thus, our experimental model is suitable to study the role of RANKL, OPG, and RANKL/OPG ratio in the pathogenesis of juxta-articular bone loss in chronic arthritis.

Therefore, we carried out an *in vivo *study to explore the potential effect of cartilage-synthesized RANKL on juxta-articular bone loss associated with RA.

## Materials and methods

### Animals

Studies were carried out in 18 adult male New Zealand rabbits with a body weight of 3.0 to 3.5 kg (Granja San Bernardo, Navarra, Spain). Animal handling and experimentation were performed in accordance with Spanish regulations and the Guidelines for the Care and Use of Laboratory Animals drawn up by the National Institutes of Health (Bethesda, MD, USA). The experimental protocol was approved by the Institutional Ethics Committee. All rabbits were allowed to adapt to the facilities for one week and were then randomly separated into three groups: 1) eight healthy rabbits (healthy group), 2) three rabbits with OA (OA group), and 3) seven rabbits with chronic antigen-induced arthritis (AIA group).

### Experimental model of chronic arthritis

**Figure 1:**
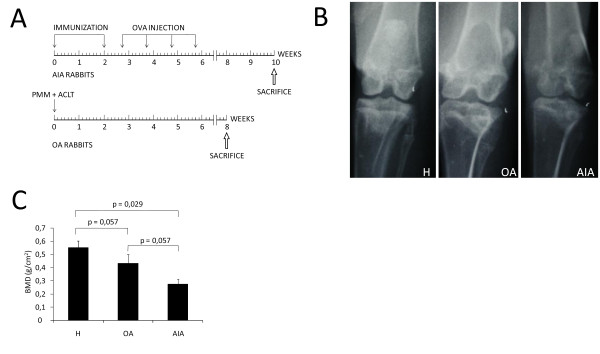
**Schematic representation of experimental models and evaluation of juxta-articular bone loss**. **(A) **Antigen-induced arthritis (AIA) was induced in seven rabbits by immunization and following weekly intra-articular knee injections of ovalbumin (OVA). Surgical osteoarthritis (OA) was induced in the knees of three rabbits by partial medial meniscectomy (PMM) and transection of the anterior cruciate ligament (ACLT). (**B) **Representative antero-posterior radiography of the knees in each group of rabbits. (**C) **Densitometric analysis of subchondral bone mineral density (BMD, g/cm^2^) in each group of rabbits. The results are expressed as mean ± standard deviation (SD) for healthy rabbits (H), and rabbits with osteoarthritis (OA) and chronic antigen-induced arthritis (AIA).

AIA was induced in rabbits as previously described in detail [31]. Briefly, animals were given two intradermal injections of 4 mg ovalbumin (OVA; Sigma-Aldrich, St. Louis, MO, USA) in Freund's complete adjuvant (Difco, Detroit, MI, USA). Beginning five days after the second injection, 1 ml of OVA (5 mg/ml in 0.9% NaCl) was injected into the knee joints on a weekly basis over the following four weeks. The animals were euthanized at the end of this period to evaluate chronic damage and not acute damage (Figure 1A). Samples of the synovial membrane, articular cartilage, and subchondral bone in each knee were collected for further studies.

### Experimental model of OA

In order to establish a control for rheumatic disease that occurs with a lower inflammatory response and a lower subchondral bone loss than chronic arthritis, surgical OA was induced in the knees of rabbits following a previously described protocol [32]. Consequently, partial medial meniscectomy and anterior cruciate ligament (ACL) sectioning were performed. The rabbits were euthanized at the end of the eighth week (Figure 1A), and knee joint tissues were obtained for further studies.

### Radiographic analysis and BMD measurements

To determine the presence of juxta-articular osteoporosis in rabbit knees, radiographic analysis and BMD measurements were taken just before the mice were euthanized. In the healthy group, these assessments were carried out at week 9 of the study.

Digital x-ray images of the knee were obtained from rabbits placed in the supine decubitus position. All radiographs were performed by the same operator using the Philips Diagnost 93 system (Philips Medical Systems, Eindhoven, The Netherlands), at 60 kV, 200 mA, 100 ms (Konica Minolta Medical film 27.9 × 35.6 cm; Casette Regius RC-110, Konica Minolta, USA). Radiographs were obtained using a vertical x-ray beam centered over the femorotibial joints and collimated from the mid-femur to the mid-tibia [33].

To determine BMD, all rabbits underwent dual-energy x-ray absorptiometry (DXA) according to previously reported work [34]. DXA was carried out using a Hologic QDR-1000/WTM pencil-beam densitometer with a 1 mm diameter collimator on the x-ray output (Hologic Inc., Waltham, MA, USA). Briefly, BMD was measured at the left knee joint with animals placed in the supine decubitus position. Four subarticular regions, each 0.06 cm^2^, corresponding to the medial and lateral femoral condyles and tibial plateaus, were selected. These regions were located, respectively, 1 mm above and below the joint line at the areas of maximum contact between the femoral condyles and tibial plateaus. The mean BMD values were considered representative of subchondral bone.

### Western-blot analysis

Tissues from synovial membranes, cartilage, and tibial subchondral bone were homogenized in liquid nitrogen, and total protein was extracted from the resulting powder by a protein extraction buffer containing 15 mM HEPES, 10% glycerol, 0.5% NP-40, 250 mM NaCl, 1 mM EDTA, 1:1000 PMSF, and a protease inhibitor cocktail (Sigma-Aldrich, St Louis, MO, USA). Protein determination was carried out as described previously [35], and subsequently 20 μg of total protein from each tissue was resolved on 15% acrylamide-SDS gels. After transfer to polyvinylidene difluoride (PVDF) membranes (Millipore, Molsheim, France) in 48 mM Tris, 39 mM glycine, and 20% methanol at 20 V for 1 h at room temperature, membranes were blocked in 5% skimmed milk in PBS-Tween 20 for 1 h at room temperature and incubated overnight at 4°C with anti-RANKL antibodies (Peprotech, Neuilly-Sur-Seine, France) and OPG (R&D systems, Minneapolis, MN, USA) at 1/1000 dilution each. Antibody binding was detected by enhanced chemoluminescence using peroxidase-labelled secondary antibodies, and the results were expressed as arbitrary densitometric units (AU). Loading control was performed on 15% acrylamide-SDS gels by employing EZBlue' Gel Staining Reagent (Sigma-Aldrich, St Louis, MO, USA).

In addition, the total amount of RANKL resulting from the sum of cartilage, synovium, and subchondral bone expressions was considered as the RANKL global expression in joint tissues. Thus, RANKL global expression was estimated as 100% and the contribution of each joint tissue was estimated in partial percentages.

### Cartilage and subchondral bone histology

After sacrifice, femur sections were fixed in 4% paraformaldehyde and further decalcified for 4 weeks in a solution made up of 10% formic acid plus 5% paraformaldehyde. The decalcified knee joints were cleaved in a sagittal plane along the central portion of the articular surface of each medial femoral condyle corresponding to the weight-bearing area, and were then embedded in paraffin block. Sections of 4 μm were stained with safranin-O Fast Green to assess pathological changes in cartilage. These samples were evaluated using a modified version of Mankin's grading score system. The Mankin score has been used by other authors to analyze cartilage damage in arthritic samples [36]. Thus, partial scores evaluating structure abnormalities, cellularity, and matrix staining (tidemark integrity was omitted) were calculated and further combined to give a maximum total score of 21 for each histological section. Each cartilage sample was evaluated twice by two experienced observers, and the mean of two reading scores was used in all statistical analyses. Samples were presented to observers in random order. The observers were blinded with respect to each other's reading, and rabbit group. Assessment of cartilage histology was performed at the weight-bearing surface of the medial femoral condyle because it shows the earliest and most severe histological abnormalities [37].

**Figure 2:**
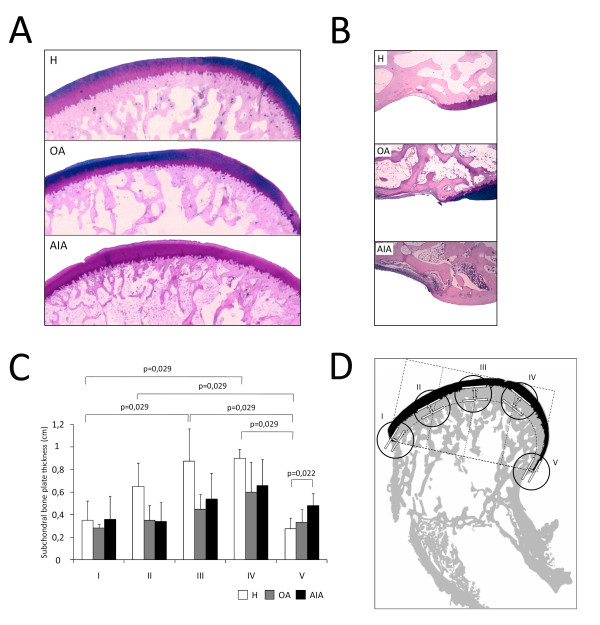
**Histological evaluation of juxta-articular bone thickness**. **(A) **Representative sections of subchondral bone stained with Alcian blue-PAS from weight-bearing regions in each group (original magnification ×100). (**B) **Representative sections of pannus-associated regions in each group (original magnification ×100). (**C) **Densitometric analysis of subchondral bone plate thickness in each group of rabbits. The results are expressed as mean ± standard deviation (SD). (**D) **Schematic view of subchondral bone measurements performed in five regions of interest of the femoral condyle in healthy rabbits (H), and rabbits with osteoarthritis (OA) and chronic antigen-induced arthritis (AIA).

Other sections were stained with Alcian blue-PAS and photographed at 4× magnification to evaluate subchondral bone plate thickness. The subchondral bone plate thickness was assessed in five different regions of the femoral condyle, including the pannus-bone interface (regions I and V), subchondral bone under the weight-bearing femoral surface (regions III and IV; Figure 2A), and subchondral bone close to the anterior synovial-bone interface (region II; Figure 2B). A straight line linking both pannus-bone interfaces was divided into five equal fragments to delineate the regions of interest. Then, two tangential lines were drawn: the first at the intersection between articular cartilage and the initial cortical layer of the subchondral bone, and the second at the point where the subchondral bone ends. The distance between these two lines was taken to define the subchondral bone plate thickness in the five regions (Figure 2D).

### Immunohistochemical localization of RANKL and OPG

Paraffin-embedded femur sections of 4 μm were prepared to detect the distribution pattern of cells expressing RANKL and OPG. The sections were incubated with antibodies against OPG (R&D Systems, Minneapolis, MN, USA) and RANKL (Santa Cruz Biotech, Heidelberg, Germany). The antibodies were detected with a biotinylated donkey anti-goat immunoglobulin G (IgG) and visualized with horseradish peroxidase/ABComplex using 3,3'-diaminobenzidine tetrahydrochloride as the chromogen (Dako, Camarillo, CA, USA). The tissues were counterstained and mounted in DPX (VWR International Ltd, Poole, England). The negative controls involved incubation with an IgG isotype.

### Cell culture

Following procedures approved by the local Ethical Committee, chondrocytes were isolated from the joint cartilage of OA patients who had undergone knee replacement surgery in the Orthopedic Surgery Department of Fundación Jiménez Díaz. Written informed consent was obtained from all patients. The chondrocytes were obtained after sequential digestion with trypsine (Lonza Group Ltd, Basel, Switzerland) for 15 minutes and collagenase type IV (Sigma-Aldrich, St Louis, MO, USA; 1 g/l) for 6 h, both at 37°C. The chondrocytes were grown to confluence in DMEM (Lonza Group Ltd, Basel, Switzerland) supplemented with 10% fetal calf serum, 60 U/ml penicillin, 60 μg/ml streptomycin, and 2 mmol/l glutamine at 37°C in the presence of 5% CO_2_. Simultaneously, peripheral blood mononuclear cells (PBMCs) were isolated from healthy donors by Ficoll gradient cell separation (Histopaque - 1077; Sigma-Aldrich, St. Louis, MO, USA). PBMCs were cultured for 24 h at a density of 2 million cells/well in 6 well-plates in α-MEM supplemented with 10% fetal calf serum, 60 U/ml penicillin, 60 μg/ml streptomycin, 2 mmol/l glutamine, and 40 ng/ml of M-CSF (Sigma-Aldrich, St. Louis, MO, USA) at 37°C in the presence of 5% CO_2._

Subsequently, confluent chondrocytes were re-suspended in α-MEM supplemented with 10% fetal calf serum and co-cultured with PBMCs at a density of 90,000 cells/well for 21 days in the presence of 40 ng/ml M-CSF (Sigma-Aldrich, St. Louis, MO, USA) and where indicated, 10^-6 ^M PGE_2 _(Cayman Chemical, Ann Arbor, MI, USA). Two osteoclastogenic inhibitors, anti-RANKL antibody (Peprotech, Neuilly-Sur-Seine, France) and OPG recombinant protein (Peprotech, Neuilly-Sur-Seine, France) were used to block osteoclast formation in the presence of 10^-6 ^M PGE_2 _in co-cultures. Both the medium and differentiation factors were changed every 48 h. After 21 days of differentiation, osteoclast differentiation was evaluated by staining for tartrate-resistant acid phosphatase (TRAP) using a commercial kit (Sigma-Aldrich, St. Louis, MO, USA). Osteoclasts were identified by the presence of ≥ 3 nuclei and purple color.

### Statistical analysis

All statistical analyses were performed using SPSS version 17.0 software for Windows (SPSS, Chicago, IL, USA), and results were expressed as the mean ± standard deviation (SD). The data from multiple groups were compared using a Kruskal-Wallis nonparametric test, and a pairwise comparison using the Mann-Whitney test was applied when overall differences were identified. Spearman correlation coefficients were calculated. *P *values less than 0.05 were considered significant.

## Results

### Juxta-articular bone loss

Radiographic analysis showed that rabbits with AIA had juxta-articular bone loss and symmetrical joint space narrowing in the affected knees. However, knees of OA rabbits had medial joint space narrowing (Figure 1B). The low densities observed on radiographs were confirmed by analyzing subchondral BMD in the knee bones (Figure 1C). In fact, rabbits with AIA had significantly lower BMD than healthy ones (0.27 ± 0.04 vs. 0.55 ± 0.05 g/cm^2^, *P *= 0.029). Furthermore, rabbits with OA also had a stronger tendency for lower BMD than healthy ones, although the difference was not statistically significant (0.43 ± 0.06 vs. 0.55 ± 0.05 g/cm^2^, *P *= 0.057). We also observed that BMD was lower in rabbits with AIA than in those with OA, though with limited statistical significance.

Juxta-articular bone loss observed in AIA rabbits was confirmed in histological studies. Indeed, thinner and discontinuous bone trabeculae are seen in AIA rabbits when compared with healthy ones (Figure 2A). To test if that bone loss was higher in the zones of bone invaded by pannus, the subchondral bone plate thickness was evaluated in weight-bearing regions and in bone-pannus interfaces. Our results showed a greater thickness in weight-bearing regions than in bone-pannus interfaces in the healthy group, whereas, in the AIA group, subchondral bone plate thickness loss was homogeneous along the condylar surface (Figure 2C). In addition, while all AIA rabbits had significant pannus associated with high osteoclastogenic activity, none of OA animals presented with pannus or bone erosions. Furthermore, pannus and bone erosions were only seen in the synovial membrane-cartilage interface of AIA animals, but not throughout the remainder of the osteochondral junction, as shown in Figure 2A and 2B.

### Histological cartilage damage

**Figure 3:**
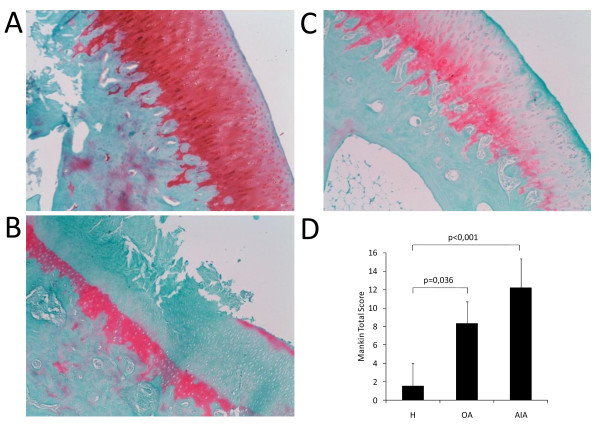
**Histological evaluation of cartilage damage**. Representative sections from healthy **(A)**, osteoarthritic **(B) **and chronic arthritic rabbits **(C) **stained with safranin-O Fast Green (original magnification ×100). **(D) **Bar graph showing the results of modified Mankin score evaluation carried out at the weight-bearing area of the medial femoral condyles. The results are expressed as mean ± standard deviation (SD) for healthy rabbits (H), and rabbits with osteoarthritis (OA) and chronic antigen-induced arthritis (AIA).

Cartilage damage in the knees of our experimental models was evaluated using a modified histopathological Mankin grading score [34]. This assessment was performed by two blinded observers, and a high correlation between both evaluations was obtained (r = 0.95). Cartilage samples from AIA and OA rabbits showed total Mankin scores that were significantly higher than those from healthy animals (AIA, 12.21 ± 3.15 vs. healthy 1.56 ± 2.49, *P *< 0.001; OA, 8.33 ± 2.36 vs. healthy 1.56 ± 2.49, *P *= 0.036), as seen in Figure 3. Furthermore, cartilage damage was inversely related to subchondral BMD in all rabbits (r = -0.829, *P *= 0.021; see Figure S1 in Additional file 1).

### RANKL and OPG protein expression in healthy and pathological joint tissues

**Figure 4:**
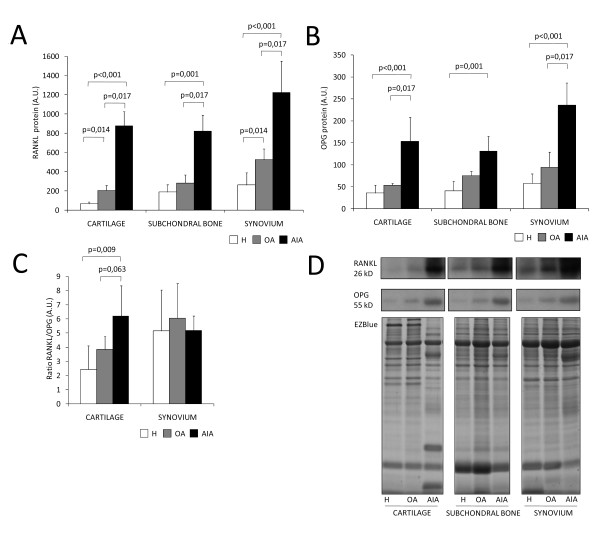
**Receptor activator nuclear factor-kappaB ligand (RANKL) and osteoprotegerin (OPG) expression in cartilage, subchondral bone and synovium**. Densitometric analysis of the expression of RANKL **(A)**, OPG **(B) **and the RANKL/OPG ratio **(C) **in cartilage, subchondral bone and synovium. **(D) **Representative western blot images for RANKL and OPG detection in cartilage, subchondral bone and synovium. EZ Blue-stained gels used as protein loading controls are also shown for healthy rabbits (H), and rabbits with osteoarthritis (OA) and chronic antigen-induced arthritis (AIA).

Our western blot results confirmed that both RANKL and OPG are expressed in healthy cartilage (Figure 4A, B). RANKL and OPG expression in AIA and OA cartilage was also analyzed. The expression of both RANKL and OPG was higher in cartilage from AIA rabbits than in cartilage from OA and healthy rabbits (RANKL in AIA, 876.80 ± 149.52 vs. OA 204.92 ± 52.47, *P *= 0.017; vs. healthy 64.15 ± 22.26, *P *< 0.001 (Figure 4A); OPG in AIA 153.79 ± 54.63 vs. OA 53.17 ± 4.61, *P *= 0.017 vs. healthy 35.63 ± 18.22 AU, *P *= 0.0001 (Figure 4B). Moreover, the RANKL/OPG ratio showed an increase in AIA cartilage when compared to healthy cartilage (6.20 ± 2.13 vs. 3.85 ± 0.90, *P *= 0.009; Figure 4C). An increased ratio, however, was not found in synovium (5.21 ± 1.02 vs. 5.14 ± 2.90, *P *= 0.613; Figure 4C) or subchondral bone (6.56 ± 1.59 vs. 5.88 ± 4.18, *P *= 0.397) of AIA rabbits when compared with healthy ones. In addition, the RANKL/OPG ratio in articular cartilage was not different from that in synovium in the AIA group (6.21 ± 2.14 vs. 5.21 ± 1.02, *P *= 0.902). RANKL expression and the RANKL/OPG ratio in articular cartilage were inversely related to subchondral BMD (RANKL: r = -0.891, *P *< 0.001; RANKL/OPG: r = -0.736, *P *= 0.01; see Figures S2 and S3 in Additional file 1).

We also analyzed the relative contribution of each tissue to the articular global RANKL production, and we observed that 30% of the total RANKL expressed in an arthritic knee is produced by the inflamed cartilage, a tissue with a poor cellular content compared to synovium or subchondral bone (data not shown).

### Localization of RANKL and OPG in articular cartilage

**Figure 5:**
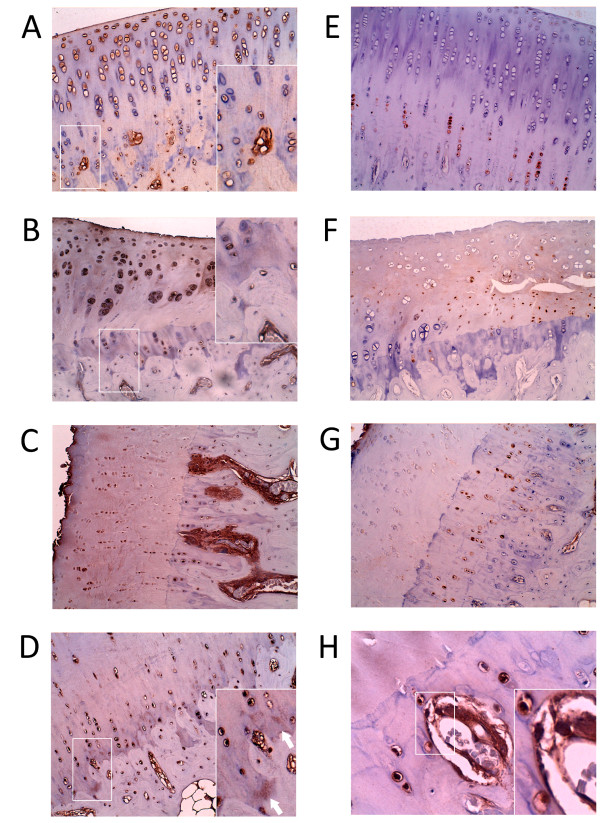
**Receptor activator nuclear factor-kappaB ligand (RANKL) and osteoprotegerin (OPG) distribution patterns in cartilage**. RANKL staining is shown in **(A) **healthy control cartilage, **(B) **osteoarthritic cartilage with insets displaying RANKL at the osteochondral junction (magnifications ×200 and ×400, respectively), and **(C) **chronic arthritic cartilage (original magnification ×200). Detail of extracellular expression of RANKL **(D)**, and its location near the blood vessel **(H)**. Insets show RANKL expression at the osteochondral junction (original magnifications ×200 and ×400, respectively). OPG staining is shown in healthy control cartilage **(E)**, osteoarthritic cartilage **(F) **and arthritic cartilage **(G) **(original magnification × 200).

Immunohistochemical detection confirmed western blot results, which showed a higher expression of RANKL (Figures 5C, D, H and Additional file 2, Figure 1) and OPG (Figure 5G), throughout the articular cartilage in AIA rabbits. In healthy cartilage, the RANKL expression was intracellular and was higher in both superficial and middle zones than in deep cartilage. In the calcified cartilage of these healthy rabbits, a mild expression of RANKL was again appreciable, and most RANKL+ chondrocytes were located around mesenchymal structures (Figure 5A). The same RANKL distribution pattern, though with a higher intensity, was gradually observed in the articular cartilage from OA (Figure 5B) and AIA rabbits (Figures 5C, D, H; see Figure S1 in Additional file 2). In the AIA rabbits, we observed that higher cartilage damage corresponded with a higher RANKL expression. RANKL expression was predominantly intra-cellular. However, a clear extracellular expression of RANKL was also detected in AIA rabbits (Figure 5D), especially near the vessel in the calcified cartilage (Figure 5H). Regarding OPG expression in healthy samples, it was only observed within chondrocytes from the deep zone (Figure 5E). The localization was almost the same for OA (Figure 5F) and AIA rabbits (Figure 5G), although we observed an increase in the intensity of the staining.

### RANKL expressed by chondrocytes has osteoclastogenic activity

**Figure 6:**
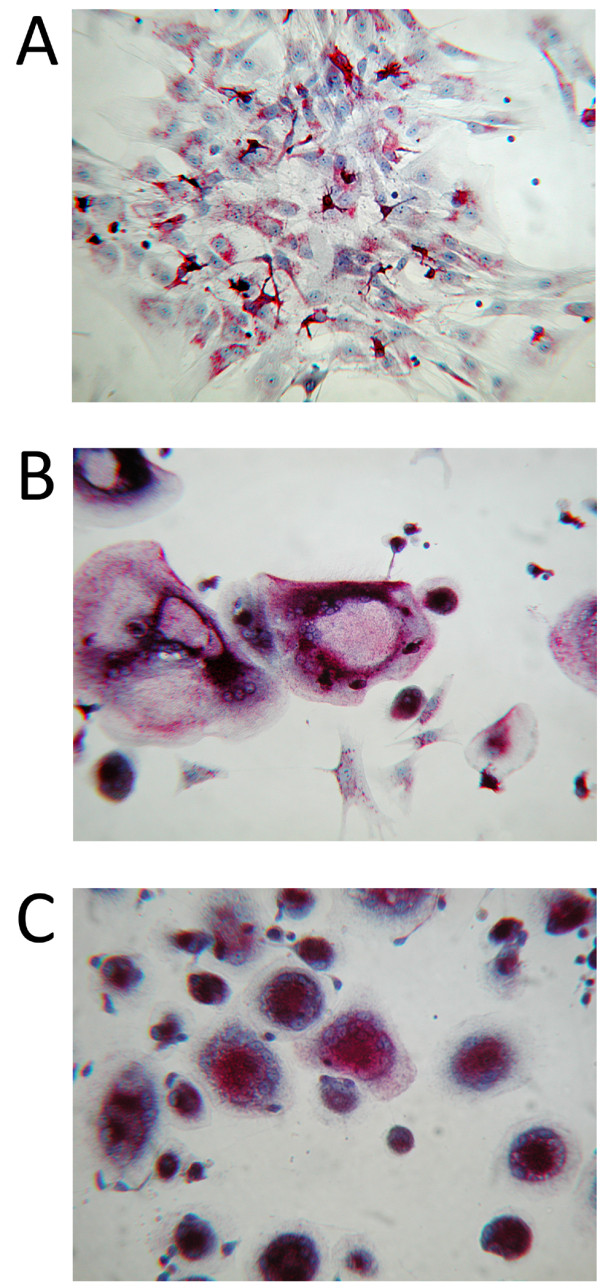
**Co-cultures of human peripheral blood mononuclear cells (PBMCs) and chondrocytes**. Images of co-cultures of human PBMCs and chondrocytes after 21 days of differentiation stimulated by macrophage-colony stimulating-factor (M-CSF) in the absence **(A) **or presence **(B, C) **of 10^-6^M PGE_2 _(original magnification ×100 and ×200, respectively). Osteoclast differentiation was evaluated by tartrate-resistant acid phosphatase (TRAP) staining, and osteoclasts were identified by the presence of ≥ 3 nuclei and purple cells.

Previous studies by our group have demonstrated that PGE_2 _induces RANKL expression by chondrocytes *in vitro *[28]. Accordingly, and in light of the increased activity of PGE_2 _in RA, we stimulated chondrocytes with PGE_2 _to replicate *in vitro *micro-environmental conditions occurring in the RA joint. Six experimental co-culture conditions were developed: 1) co-culture of PBMCs with chondrocytes in the presence of M-CSF; 2) co-culture of PBMCs with chondrocytes in the presence of M-CSF and PGE_2_; 3) co-culture of PBMCs with chondrocytes in the presence of M-CSF, PGE_2 _and anti-RANKL antibody; 4) co-culture of PBMCs with chondrocytes in the presence of M-CSF, PGE_2 _and OPG recombinant protein; 5) culture of PBMCs in the presence of M-CSF and PGE_2_, and 6) culture of PBMCs in the presence of M-CSF and RANKL. Our results demonstrated that basal RANKL production by chondrocytes was not enough to induce osteoclastogenesis from monocytes *in vitro *(Figure 6A). However, RANKL produced by PGE_2_-stimulated chondrocytes induced differentiation of monocytes into osteoclasts (Figure 6B and 6C). In addition, we have observed that both RANKL inhibitors blocked osteoclastogenesis as no TRAP+ cells were seen in these two culture conditions (data not shown).

## Discussion

In this study, we have shown that the expression of both RANKL and OPG were higher in the articular cartilage of rabbits with AIA than in healthy cartilage. The localization of RANKL in knee cartilage was also different between AIA and healthy cartilage. While both intracellular and clear extracellular RANKL signals were observed in rabbits with AIA, especially around the mesenchymal structures in the calcified cartilage, healthy rabbits showed mild intracellular and no extracellular RANKL signals in this area. In addition, the resorptive signal measured by the RANKL/OPG ratio was increased in the articular cartilage of AIA rabbits, and this increase was simultaneous to a significant bone loss in the subchondral plate that was homogeneous along the femoral condyle surface.

Systemic bone loss in RA is a multifactorial and complex alteration, in which chronic inflammation plays an important role. Within the joint, the focal bone erosions and juxta-articular bone loss are observed at different localities. Focal bone erosions are mainly observed in the marginal zones where pannus invades the cortical bone, whereas juxta-articular bone loss occurs in the subchondral trabecular bone [9]. Whether these different localities are related to different molecular mechanisms remains to be elucidated. However, it seems clear that a direct synovial-bone contact exists in the sites where focal bone erosions have been described. Bone loss processes are mainly due to an increased number of osteoclasts [10], which is mainly regulated by the OPG/RANK/RANKL system [13]. Several studies focused on the expression of RANKL in arthritic tissues have shown that RANKL is increased in the inflamed synovium of patients with RA [20,38,39]. Furthermore, synovial RANKL expression was mainly localized in focal areas of pannus where an invasion of the subchondral bone was observed [40], and this localization coincides with the sites where osteoclast precursors have been identified [20]. Therefore, RANKL would be able to differentiate mononuclear cells into osteoclast-activating bone erosions. These data suggest that the RANKL produced by synovial cells contributes to RA-associated bone erosion.

According to our data, subchondral bone loss in AIA rabbits was not higher in the zones where direct contact between the bone and the synovial membrane took place. On the contrary, subchondral bone loss was uniform along the AIA subchondral plate, and was similar at the pannus-bone interface margins and at the weight-bearing regions. So, it is tempting to speculate that synovial membrane is not the sole factor responsible for this specific bone lesion. Within the joint, hyaline chondrocytes are also able to synthesize RANKL [28]; however, scarce attention has been given to its potential role in RA-related subchondral alterations. According to these observations, we hypothesize that the RANKL synthesized in cartilage, a tissue intimately close to the subchondral bone surface, might have a paracrine effect modulating subchondral bone remodelling during RA. Although it has been reported that the synovial membrane may be the major source of RANKL [20], we observed that, in AIA rabbits, articular cartilage synthesizes about 30% of total RANKL produced in the arthritic joint. In addition, we found a similar RANKL/OPG ratio in cartilage and in the synovial membrane, indicating that both tissues would render an equally potent resorptive signal. We have demonstrated an increase in RANKL immunostaining in AIA cartilage as compared to OA and healthy cartilage, and remarkably the increase of RANKL/OPG ratio in the cartilage of AIA rabbits was simultaneous to subchondral bone loss. Furthermore, rabbits with lesser subchondral bone loss, such as the OA rabbits in our study, also showed lower resorptive signal in the cartilage. In contrast, although RANKL expression increases accordingly with the degree of synovial inflammation in pannus-associated regions, the RANKL/OPG ratio remained similar in all three rabbit groups.

Until now, no data have been published demonstrating a direct effect on mature chondrocytes of the RANKL synthesized in cartilage, although scarce approaches have been made available in the literature. In this sense, it is also unknown whether a putative effect of RANKL in chondrocytes would be mediated by its receptor RANK or whether the levels of RANK are sufficient to transduce the RANKL signal [16]. On the contrary, a variety of studies suggest that RANKL might have a paracrine effect acting on the subchondral bone [41-43]. We observed that the increase in the RANKL expression in the cartilage of AIA rabbits was linked to the presence of extracellular RANKL in the calcified cartilage of these rabbits. Previous results from our group have also shown that RANKL is localized in the extracellular matrix of cartilage in human OA and could reach the subchondral bone though the calcified cartilage [28]. These results point out that the RANKL synthesized by chondrocytes might act on subchondral bone cells stimulating juxta-articular bone loss. Our observation is also in line with previous data which demonstrate that soluble RANKL produced by hypertrophic chondrocytes is a biologically active molecule during bone growth [42,43] that acts in a paracrine manner on the subchondral bone plate [44]. We have also shown that RANKL synthesized by PGE2-stimulated mature articular chondrocytes is also biologically active and is responsible for the mononuclear cell differentiation into osteoclasts in the absence of exogenous RANKL. Indeed, osteoclast formation was blocked when PBMCs and chondrocyte co-cultures were treated with RANKL inhibitors. The ability of human synovial cells to stimulate osteoclastogenesis has been previously reported, although no data exist on the ability of human chondrocytes to induce osteoclast differentiation.

RA treatment with corticosteroids [45] or methotrexate [46] has been described to regulate bone loss, modulating RANKL expression in synovial tissue. In this context, our results suggest it would be worthwhile to study whether these pharmacologic agents or other anti-inflammatory drugs might also prevent the RA-related bone loss inhibiting the RANKL expression in cartilage.

## Conclusions

In summary, we propose a new pathological mechanism underlying the juxta-articular bone loss associated with chronic arthritis. It has been previously described that the synovial membrane is an important source of RANKL, which is highly involved in the enhanced osteoclastic activity responsible for this form of bone loss [5]. However, according to the new mechanism proposed here, pathological changes in the expression and localization of RANKL synthesized by chondrocytes might be also involved in juxta-articular bone loss. These findings represent an *in vivo *confirmation of previous anatomical [47,48] and *ex vivo *[44] studies, whose results collectively suggest that the calcified cartilage is permeable and may transport solutes with a molecular weight lower than 376 kDa.

## Abbreviations

AIA: antigen-induced arthritis; ACL: anterior cruciate ligament; AU: arbitrary densitometric units; BMD: bone mineral density; DXA: dual-energy x-ray absorptiometry; H: healthy; IgG: immunoglobulin G; IL: interleukin; M-CSF: macrophage-colony stimulating factor; OA: osteoarthritis; 1,25(OH)_2_D_3_: 1,25-dihydroxycholecalciferol; OPG: osteoprotegerin; OVA: ovalbumin; PMM: partial medial meniscectomy; PVDF: polyvinylidene difluoride; PTH: parathyroid hormone; PBMCs: peripheral blood mononuclear cells; PGE_2_: prostaglandin E_2_; RA: rheumatoid arthritis; RANKL: receptor activator nuclear factor-kappaB ligand; SD: standard deviation; TRAIL: TNF apoptosis related inducing ligand; TRAP: tartrate-resistant acid phosphatase; TNF-α: tumor necrosis alpha.

## Competing interests

The authors declare that they have no competing interests.

## Authors' contributions

All authors were involved in drafting the article or revising it critically for important intellectual content, and all authors approved the final version to be published. GH-B had full access to all of the data in the study and takes responsibility for the integrity of the data and the accuracy of the data analysis. GH-B, RL and MJM-C were involved in the study conception and design. MJM-C, IP-P, LT and RL were involved in the acquisition of data. MMJ-C, IP-P, JAR-B, RL and GH-B were involved in the analysis and interpretation of data

## Supplementary Material

Additional file 1 **Relationships between articular cartilage parameters and subchondral bone mineral density (BMD)**. Figure S1 in Additional file 1: A figure showing Spearman correlation between cartilage damage and subchondral BMD. Figure S2 in Additional file 1: A figure showing Spearman correlation between receptor activator nuclear factor-kappaB ligand **(**RANKL) protein expression in articular cartilage and subchondral BMD. Figure S3 in Additional file 1: A figure showing Spearman correlation between RANKL/osteoprotegerin (OPG) ratio in articular cartilage and subchondral BMD. Click here for file

Additional file 2 **Figures showing the receptor activator nuclear factor-kappaB ligand (RANKL) distribution pattern in cartilage**. Figure S1 in Additional file 2: A figure showing extracelullar expression of RANKL in the rabbits with antigen-induced arthritis (AIA). Figure S2 in Additional file 2: A figure showing negative control of RANKL expression in the same area (original magnifications ×400). Click here for file
