# Supplementary figures and images for: RANKL synthesized by articular chondrocytes contributes to juxta-articular bone loss in chronic arthritis
Source: Arthritis Res Ther. 2012 Jun 18;14(3):R149. doi: 10.1186/ar3884 (PMC3446534; doi:10.1186/ar3884)

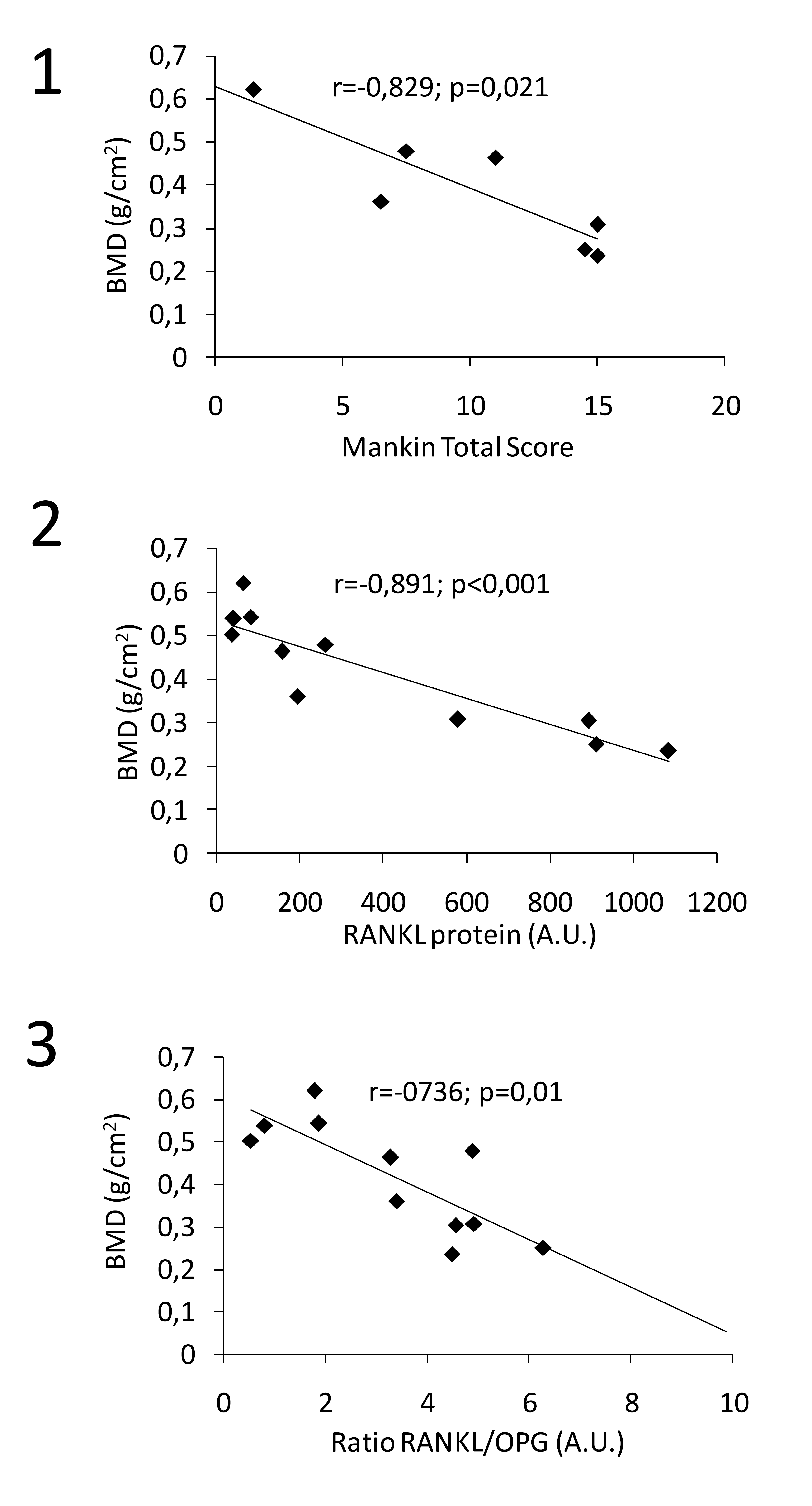

Supplement: Additional file 1 — Relationships between articular cartilage parameters and subchondral bone mineral density (BMD). Figure S1 in Additional file 1: A figure showing Spearman correlation between cartilage damage and subchondral BMD. Figure S2 in Additional file 1: A figure showing Spearman correlation between receptor activator nuclear factor-kappaB ligand (RANKL) protein expression in articular cartilage and subchondral BMD. Figure S3 in Additional file 1: A figure showing Spearman correlation between RANKL/osteoprotegerin (OPG) ratio in articular cartilage and subchondral BMD. [file ar3884-S1.TIFF]

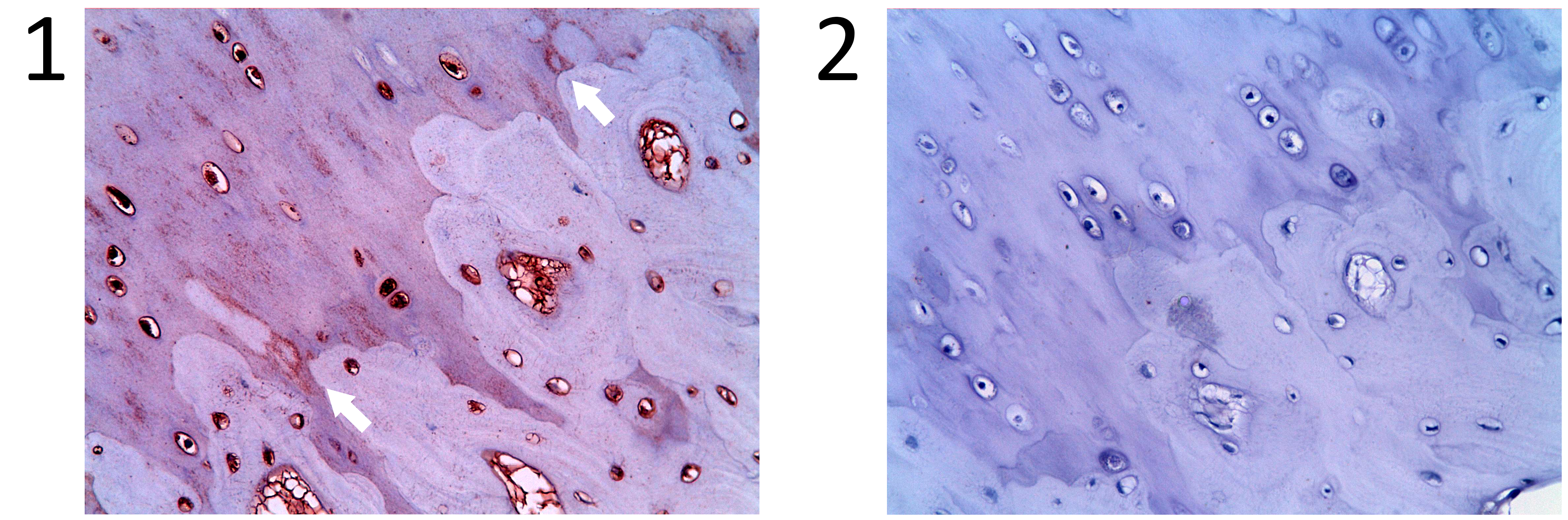

Supplement: Additional file 2 — Figures showing the receptor activator nuclear factor-kappaB ligand (RANKL) distribution pattern in cartilage. Figure S1 in Additional file 2: A figure showing extracelullar expression of RANKL in the rabbits with antigen-induced arthritis (AIA). Figure S2 in Additional file 2: A figure showing negative control of RANKL expression in the same area (original magnifications ×400). [file ar3884-S2.TIFF]
